# STAT3-Mediated Transcriptional Regulation of Osteopontin in STAT3 Loss-of-Function Related Hyper IgE Syndrome

**DOI:** 10.3389/fimmu.2018.01080

**Published:** 2018-05-17

**Authors:** Shubham Goel, Smrity Sahu, Ranjana W. Minz, Surjit Singh, Deepti Suri, Young M. Oh, Amit Rawat, Shobha Sehgal, Biman Saikia

**Affiliations:** ^1^Department of Immunopathology, Postgraduate Institute of Medical Education and Research, Chandigarh, India; ^2^Department of Pediatrics, Advanced Pediatrics Centre, Postgraduate Institute of Medical Education and Research, Chandigarh, India; ^3^Cell Line Development Team, Bio Research Institute, Genexine Inc, Seongnam, South Korea

**Keywords:** craniosynostosis, dental anomalies, hyper-IgE syndrome, osteopontin, STAT3 signaling, skeletal anomalies

## Abstract

**Background:**

Hyper-IgE syndrome (HIES) caused by loss-of-function (LOF) mutations in STAT3 gene (STAT3 LOF HIES) is associated with dental and facial abnormalities in addition to immunological defects. The role of STAT3 in the pathogenesis of the dental/facial features is, however, poorly elucidated.

**Objectives:**

Since mechanism of cellular resorption of mineralized tissues such as bone and teeth are similar, we attempted to study the expression of genes involved in bone homeostasis in STAT3 LOF HIES.

**Methods:**

Peripheral blood mononuclear cells from healthy controls (HCs), STAT3 LOF HIES patients, STAT3^−/−^ PC-3 cells and STAT3^+/+^ LNCaP cells were stimulated with IL-6 and quantitative PCR array was performed to study the relative mRNA expression of 43 pre-selected genes. PCR array finding were further evaluated after *stattic* induced STAT3 inhibition.

**Results:**

Osteopontin (OPN) gene was seen to be significantly upregulated after IL-6 stimulation in HC (mean fold change 18.6, *p* = 0.01) compared with HIES subjects. Inhibition of STAT3 signaling by *stattic* followed by IL-6 stimulation abrogated the OPN response in HCs suggesting that IL-6-induced STAT3 signaling regulates *OPN* expression. Bioinformatics analysis predicted the presence of STAT3 response element TTCCAAGAA at position -2005 of the OPN gene.

**Conclusion:**

Regulation of OPN gene through IL-6-mediated STAT3 activation and its significant dysregulation in STAT3 LOF HIES subjects could make OPN a plausible candidate involved in the pathogenesis of dental/facial manifestations in HIES.

## Introduction

Hyper-IgE syndrome (HIES) associated with loss-of-function (LOF) mutations in the STAT3 gene (STAT3 LOF HIES) (1, 2) with resultant deficiency of TH17 cells and its secreted cytokine IL-17 (3–5), are now known to underlie majority of the sporadic and autosomal dominant forms of HIES. The syndrome is characterized by varying degrees of immunodeficiency along with dental, connective tissue, and skeletal defects. Features include high-serum levels of IgE (>2,000 IU/ml), staphylococcal skin abscesses, recurrent bacterial infections, pneumonia with *pneumatocele* formation, mucocutaneous candidiasis, and chronic eczematoid dermatitis manifesting early in life. Coarse facies and mild eosinophilia are variable features (6–10). Though most of the features of autosomal dominant STAT3 LOF HIES reflect a deficient immune system, the non-immunological features *viz*. dental abnormalities (retained primary teeth, non-eruption of permanent teeth, double rows of teeth), anomalies in midline facial development (prominent forehead, wide nasal bridge, broad nose, prominent mandible), and skeletal abnormalities (bone fractures, hyperextensible joints, scoliosis, and craniosynostosis) reflects the multisystem nature of the disease (1, 6, 11–14). The exact mechanism by which the STAT3 defect leads to the dental/craniofacial manifestations is, however, not clearly defined. Recent description of a HIES phenotype with craniosynostosis, and scoliosis in mutations of the IL6ST gene encoding the gp130 receptor, and of isolated craniosynostosis, maxillary hypoplasia, delayed tooth eruption, supernumerary teeth, and minor digital anomalies in IL-11RA mutations (15, 16), strongly suggests involvement of the IL-11/gp130/STAT3 axis in craniofacial and dental features.

It is known that mechanisms in cellular resorption of bone and teeth are similar (17) and delayed shedding of primary teeth is essentially a manifestation of ineffective apical root resorption. On similar lines, the steady state in mature cranial and facial bone sutures represents a balance between osteogenesis and resorption (18). Osteoclasts (OC) and odontoclasts (OdCs) are crucial for resorption of mineralized tissues of the craniofacial complex and the rate of resorption as well as the timing of OC/OdC function during pre-emergent tooth eruption is crucial to support the erupting tooth to move along the appropriate path. Though the precise mechanism of apical root resorption in shedding of primary teeth have been elusive, decreased OdC activity is observed in over-retention of primary teeth (19). Though the maintenance of cranial sutures is genetically much more complex, similar mechanisms of OC function and resorption are thought to be involved (18). Minegishi et al. in an attempt to correlate STAT3 with OC differentiation and function observed that OCs from individuals with STAT3 LOF HIES tend to show higher resorptive activity compared with controls without any effect on OC differentiation (2). While this might explain the skeletal features of osteoporosis and fractures (increased OC activity), it fails to explain retention of teeth and craniosynostosis (decreased OC activity) in HIES. A similar dissociation is observed in the IL-11RA defect and craniosynostosis, as IL-11 is known to have a stimulatory effect in osteoblasts predicting osteopenia in IL-11RA deficiency, but the opposite turns out to be true (18). No detailed study has, however, been performed on OC function, osteoporosis, and fractures till date in HIES. IL-17 has also been found to be involved in bone remodeling by inducing the expression of receptor activator of nuclear factor κB ligand (RANKL) which in turn increase osteoclastogenesis causing osteoporosis (20) implying that TH17 deficiency would lead to decreased osteoclastogenesis. Defects in the IL17 axis, however, are known to cause chronic mucocutaneous candidiasis (21, 22), which do not show any involvement of the dental/skeletal system whatsoever and hence IL17 deficiency as the cause for the dental/skeletal defects in HIES seems unlikely.

The explanation behind the dental/facial involvement in STAT3 LOF HIES would probably lie on a molecule that has both immune and osteogenic/odontogenic functions and is regulated through STAT3. In the light of the above facts, the study was aimed to evaluate plausible molecules involved in bone homeostasis that could be dysregulated in STAT3 LOF HIES.

## Materials and Methods

### Study Design and Subjects

This prospective study was carried out in the department of Immunopathology, Postgraduate Institute of Medical Education and Research, Chandigarh, India. Study protocol was approved by the Institute Ethics Committee (IEC No. 8827-PG 10-1TRG/8450). All subjects were recruited after obtaining a written informed consent prior to enrollment as per IEC guidelines.

Three STAT3 LOF HIES subjects with mutation in STAT3 DNA binding domain (P1; 35 years male; Exon 10, A>C; g.54792; c.1018; p.K340Q), transactivation domain (P4; 3 years male; Exon 22, C>T; g.71311; c.2141; p.T714I), and SH2 domain (P5; 16 years male; Exon 21, T>C; g.66092; c.1979; p.M660T), respectively, were enrolled in the study (Table 1). The clinical details and laboratory investigations of the subjects P1, P4, and P5 have been previously reported (23, 24). All three STAT3 LOF HIES subjects had reduced TH17 cell numbers, high serum IgE levels, and NIH score more than 40 (Table 1). Healthy volunteers without any history of chronic illness and normal serum IgE levels were recruited as controls.

**Table 1:** Clinical and genetic profile of hyper IgE syndrome patients.

Patient ID	Age (years)^c^	Sex	NIH score	STAT3 mutation	IgE levels (IU/ml)	Eosinophilia (cells/μl)	Eczema	Skin abscesses	Pneumonia	Skeletal abnormalities^b^
P1	40	M	52	1018A>C, K340Q (DBD)^a^	103,200	295	+	+	+	1, 2
P4	3	M	42	2141C>T, T714I (TA)	5,000	948	+	−	+	1, 2
P5	16	M	49	1979T>C, M660T (SH2)^a^	9,180	968	+	+	+	1, 3, 4

*^a^Novel mutation reported by Saikia et al. (23, 24)*.

*DBD, DNA binding domain, SH2, Src homology domain; TA, transactivation domain*.

*^b^1: coarse facies; 2: increased nasal width; 3: pathological fractures; 4: retained primary teeth*.

*^c^Age of the patients at the time of analysis*.

### Sample Collection and Peripheral Blood Mononuclear Cell (PBMC) Isolation and Cell Culture

8 ml of peripheral venous blood was collected in heparin from the patients and controls for PBMC isolation using density gradient centrifugation method. Cell viability was checked by trypan blue dye (Sigma Aldrich, USA) exclusion test and more than 98% viability was obtained. PBMCs (1 × 10^6^) were suspended in RPMI 1640 without fetal bovine serum (FBS) and incubated with or without IL-6 (Peprotech, Rocky Hill, NJ, USA) at a dose of 30 ng/ml for 2 h. For STAT3 inhibition, PBMCs were incubated in the presence of 10 µM of the *stattic* (Sigma Aldrich, USA), a specific STAT3 inhibitor, for 1 h followed by treatment with 30 ng/ml of IL-6 for 2 h in 5% CO_2_. Cells were subsequently harvested for RNA extraction. Two prostate cancer cell lines viz. STAT3^−/−^ PC-3 (25, 26) and STAT3^+/+^ LNCaP (25, 27) were procured from National Center for Cell Science, Pune, India. The cells were maintained in RPMI 1640 and Ham’s F-12 (Sigma Aldrich, USA) media, respectively, supplemented with 10% FBS and antibiotics. Cells were grown as monolayers at 37°C in 5% CO_2_. Confluent cultures were trypsinized (0.25% trypsin + 0.02% EDTA), re-suspended in complete medium, and seeded at a concentration of 1–2 × 10^5^ cells/well/ml into 12-well tissue culture plates and incubated for 48 h at 37°C to obtain 70–80% confluency. Once confluent, cells were serum starved for 4 h before stimulation with IL-6 for 2 h, harvested, and RNA was extracted.

### RNA Extraction and cDNA Synthesis

Total RNA was isolated from cells using Trizol according to the manufacturer’s protocol (Ambion, Life technologies, CA, USA) and stored at −80°C. DNase treatment was carried out using RNase-free DNase kit (Sigma Aldrich, USA). RNA concentration was determined by Spectrophotometer (Bibby Scientific, Jenway, USA) by taking absorbance at 260 nm and quantity was adjusted to >40 μg/ml. Purity was checked by taking absorbance ratio of 260/280 and values of 1.9–2.0 was taken to indicate good quality RNA. cDNA synthesis was carried out using the Revert Aid™ First strand cDNA Synthesis Kit (Fermentas Life Sciences, USA) according to the manufacturer’s instructions and stored at −80°C till further use.

### Relative Gene Expression Study by Customized PCR Arrays

Forty-three genes, including transcription factors, cytokines, growth factors, negative regulators, receptors, extracellular membrane proteins, and proteases, were selected based on their involvement in bone metabolism and immune function after a careful literature search (Table 2). Functional aspects of the selected genes are illustrated in Table S1 in Supplementary Material. Arrays were customized by Molecular Diagnosis & Research Laboratories, India. Each 96-well plate composed of 43 genes (in duplicate) along with 4 housekeeping genes and negative RT-control. Relative expression of these genes was studied in PBMCs from healthy controls (HCs) (*n* = 5) and HIES patients (*n* = 3) together with LNCaP and PC-3 cells after stimulation with IL-6 for 2 h.

**Table 2:** List of genes studied by PCR array.

	Bone homeostasis related genes	Immune system related genes
Growth factors		Epigen, Epireg

Cytokines	LIF, Onco M	LIF

Negative regulators	OPG, Noggin	Blmp1, SOCS3, PIAS3

Transcription factors	RUNX2, Sp7 (Osterix), TWIST1	RORyt

Proteases	MMP2, MMP8, MMP9, Cath K	Cyst C, Cath E

Protease inhibitors	TIMP2	b3 int

Receptors	V.D3, LIFR	V.D3, LIFR, gp130, T3 zeta chain

Adhesion molecules	VCAM	b3 int, SELL, VCAM

Kinases		MAPK1, ROCK1

Phosphatases		Calcen, SHP2

Others	BSP, DKK1, BGP, DKK2, OPN (osteopontin), SPARC, LRP5	SIRT1, Cam1, FLt3L, MTTF, PLCy2

Housekeeping genes	RPL37A, RPLP1, GAPDH, ACTB	

Briefly, 5 ng template cDNA was mixed with 2× Real Time SYBR Green PCR master mix (Roche Diagnostic, Germany). Equal volumes of this mixture was added to each well of the 96-well PCR array plate containing the pre-dispensed gene-specific primer sets in duplicate, and quantitative real time PCR (qRT-PCR) carried out using Roche LC 480 real time PCR machine (Roche Diagnostic, Germany). The amplification conditions used were initial denaturation at 95°C for 10 min, followed by 40 cycles of denaturation at 95°C for 15 s, annealing at 60°C for 10 s, and extension at 72°C for 20 s. The threshold cycle (*C*_t_) values were derived for all genes on all PCR arrays. Finally, the fold-changes in gene expression were calculated using RT^2^ PCR Array analysis software, applying the 2^−^(Δ*C*_t_) formula:

$$\text{Fold Change}=\frac{2^{-}(\Delta C_{\text{t}})\text{Stim}}{2^{-}(\Delta C_{\text{t}})\text{Unstim}}$$

where Δ*C*_t_ was calculated using the formula: Δ*C*_t_ = *C*_t_(GOI) − Ave *C*_t_ (HKG) (GOI: Gene of interest, HKG: Housekeeping gene, Ave: Average).

### Relative Gene Expression Analysis by qRT-PCR

Relative expression of *SOCS3, STAT3, and OPN* genes were determined by qRT-PCR using SYBR Green chemistry. Reactions were performed in triplicates with 50 ng of cDNA in 20 µl final reaction volume containing 1× SYBR Green dye (Roche Diagnostics, Switzerland) and 10 pmol of each primer (Sigma, USA). Each sample along with calibrator was normalized with β-actin gene and the ratio of gene expression was obtained by applying 2^−^ΔΔ*C*_t_ formula: ΔΔ*C*_t_ = Δ*C*_t_ (un-stimulated) − Δ*C*_t_ (stimulated), where Δ*C*_t_ was calculated using the formula: Δ*C*_t_ = *C*_t_^GOI^ − *C*_t_^HKG^ (GOI: Gene of interest; HKG: Housekeeping gene). The primer sequences and PCR cycle conditions are mentioned in Table 3.

**Table 3:** Primer sequences and PCR conditions for *STAT3, SOCS3, OPN, and β-actin* genes.

Genes	Primer sequence (5′–3′)	Product size (bp)	Annealing Temp (30 s)	Extension time (72°C)
*STAT3*	CATATGCGGCCAGCAAAGAA	152	58°C	30 s
	ATACCTGCTCTGAAGAAACT			
*OPN*	CAGTGATTTGCTTTTGCCTCC	149	60°C	30 s
	ATTCTGCTTCTGAGATGGGTC			
*SOCS3*	CACCTGGACTCCTATGAGAAAGTCA	74	60°C	20 s
	GGGGCATCGTACTGGTCCAGGAA			
β*-Actin*	AGCACAGAGCCTCGCCTTTGC	280	60°C	40 s
	GGGGCATCGTACTGGTCCAGGAA			

### Evaluation of Phosphorylated STAT3 (p.Y705)

Phosphorylation of *STAT3* was analyzed using flow cytometry. Fresh whole blood (100 µl) or PBMCs (5 × 10^5^) from HCs and HIES subjects and cell lines (1 × 10^6^ cells) were incubated with IL-6 with or without *stattic*. Cells were simultaneously fixed and RBCs were lysed (in case of whole blood) using BD fix and lysing solution (BD Biosciences, USA). Cells were then permeabilized for 20 min using Perm III solution (BD Biosciences, USA) and subsequently incubated with Alexa Fluor 647 phospho-STAT3 antibody against phospho-Y705 (BD Biosciences, USA) for 30 min at room temperature. Washing was done twice with stain buffer (PBS + 2% FBS + 0.09% sodium azide) and cells were suspended in 1% paraformaldehyde. The cells were then acquired and analyzed in a BD FACS Aria III, flow cytometer (BD Biosciences, USA) using BD FACS Diva software (version 8).

### Serum OPN Quantification by ELISA

Estimation of serum *OPN* was done using pre-coated commercially available ELISA kit (eBiosciences, San Diego, CA, USA) as per the manufacturer’s protocol. Plate was read at 570 and 450 nm using an ELISA reader (Tecan, Germany). Standard curve range of the kit was 0.47–30 ng/ml with sensitivity of 0.26 ng/ml.

### Statistical Analysis

Statistical analysis was performed using Graph Pad Prism software version 5.0 (GraphPad Software, La Jolla, CA, USA). Skewed and normally distributed data were deduced as median and mean, respectively. Comparisons between groups of related samples were assessed by the paired or unpaired *t*-test (for normally distributed data). Wilcoxon or Mann–Whitney test were performed for non-parametric data. ANOVA test was performed for more than two group comparisons. A *p* value of ≤0.05 was considered to be significant.

## Results

### STAT3 Phosphorylation and IL-6/STAT3 Mediated SOCS3 Gene Expression in HCs, HIES Subjects, and Cell Lines (LNCaP and PC3)

**Figure 1:**
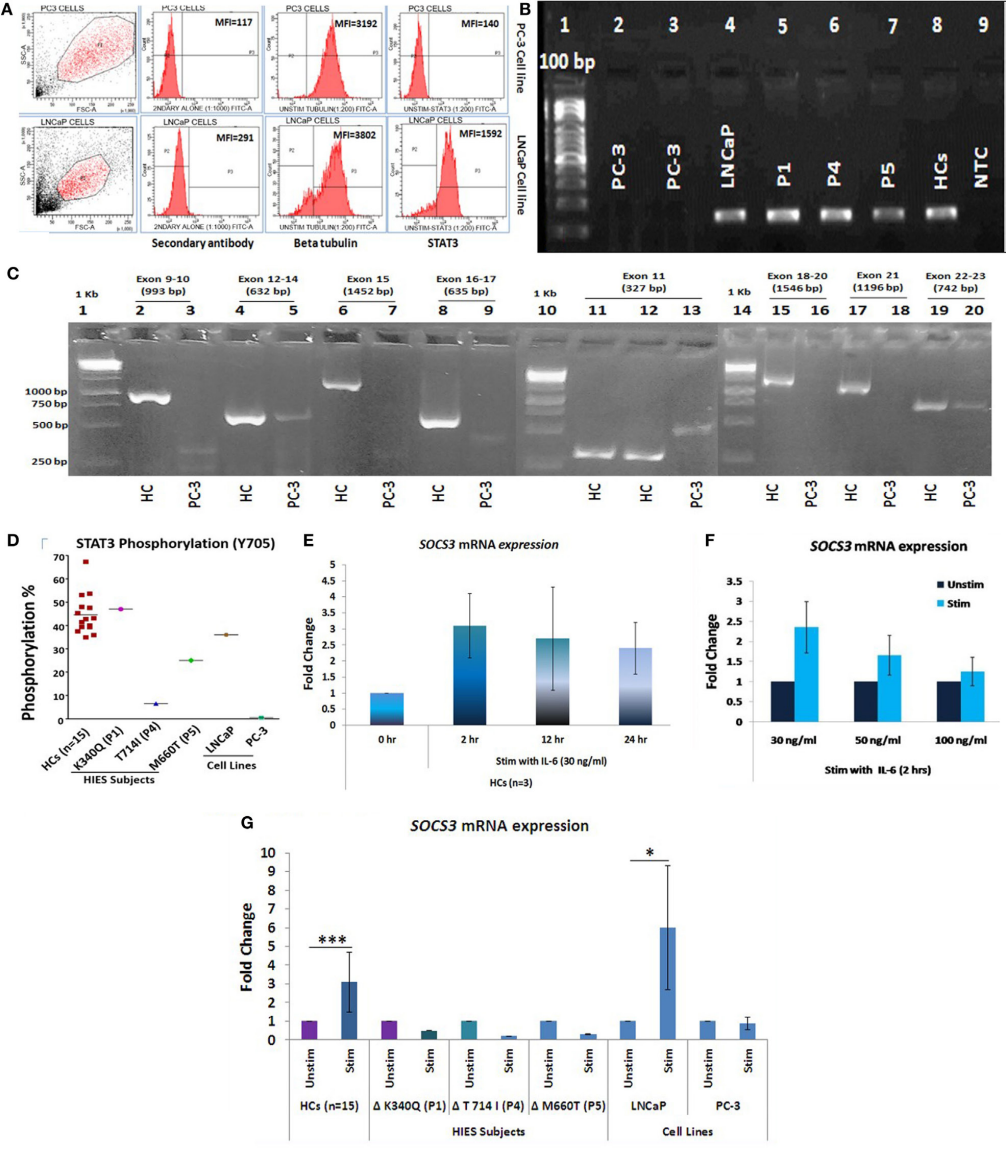
**(A)** Representative flow cytometry histogram of *STAT3* protein expression in PC-3 and LNCaP cells. PC-3 cells had no expression of *STAT3* protein. **(B)** STAT3 mRNA expression in PC3, LNCaP, hyper-IgE syndrome (HIES) subjects and healthy controls (HCs) by conventional PCR. PC-3 cells showed no expression of STAT3 mRNA. **(C)** Agarose gel pictures showing either absence or non-specific/extremely faint bands of STAT3 amplification (exons 9–22) in PC-3 cells. Gel 1 (Exons 9–10/12–24/15 and 16–17), gel 2 (exon 11), and gel 3 (exons 18-20/21/22-23) have been grouped together for representation purpose. **(D)** Scatter plot showing per cent STAT3 phosphorylation in HCs, HIES subjects, LNCaP, and PC3 cells; mean values are shown as horizontal bars. Bar graphs showing quantitative real time PCR (qRT-PCR) of *SOCS3* mRNA expression in HC peripheral blood mononuclear cells stimulated with IL-6 in a **(E)** time-dependent manner at 30 ng/ml and **(F)** dose-dependent manner for 2 h. Data are shown as mean ± SD for three independent experiments. **(G)** Bar graphs showing qRT-PCR results of *SOCS3* expression from HCs, HIES subjects, LNCaP, and PC-3 cells stimulated with IL-6. The primary transcript for each gene were assayed at least in duplicate and normalized to *β-actin* expression. (Data are represented as mean ± SD, *p < 0.05, **p < 0.01, ***p < 0.001, paired *t*-test.)

Presence or absence of *STAT3* was checked in LNCaP and PC-3 cells at protein, mRNA, and genomic level by flow cytometry, qRT-PCR, and sequencing, respectively. No *STAT3* protein or mRNA expression was observed in PC-3 cells while LNCaP showed *STAT3* mRNA as well as protein expression (Figures 1A,B). PCR and sequencing of STAT3 gene (Exon 9 to Exon 23) in PC-3 cells showed either absence or faint and/or non-specific amplicons indicating a large deletion or absence of STAT3 gene (Figure 1C). PC3 cells have been shown to lack an entire chromosomal region spanning 500 kb, which includes the entire 30 kb STAT3 gene (26). Exons 1–8 were not further examined. *STAT3* phosphorylation was absent in PC-3 cells whereas LNCaP cells showed normal (36%) phosphorylation (Figure 1D). These experiments validated the use of the LNCaP and PC3 cell lines as +ve and −ve controls for STAT3. The range of *STAT3* phosphorylation in HCs (*n* = 15) was 35–67% (mean 44.62 ± 8.5, median 42.8). P1 with mutation in DNA binding domain showed normal phosphorylation (46%) while P4 and P5 with mutations in transactivation domain and SH2 domain, respectively, had reduced STAT3 phosphorylation: 6.5 and 25% (Figure 1D).

To evaluate the IL-6/STAT3 pathway activation, mRNA expression of *suppressor of cytokine signaling 3* (*SOCS3*), a known downstream target of STAT3 (28) was checked to confirm signaling through the IL-6/STAT3 pathway. For optimizing time and dose of IL-6, experiments were performed with HC PBMCs at different time intervals (0, 2, 12, and 24 h) and various IL-6 dosage (30, 50, and 100 ng/ml) (Figures 1E,F). IL-6 at a dose of 30 ng/ml for 2 h showed maximum up-regulation of *SOSC3* mRNA using qRT-PCR and the same was used for the subsequent experiments. *SOCS3* was found to be significantly upregulated in HCs (*n* = 15, mean fold change 3.1 ± 1.6, *p* = 0.001) while all three HIES subjects showed downregulated *SOSC3* mRNA expression. LNCaP cells showed mean fold change of 6 ± 3.3 (*p* = 0.03), while PC-3 cells showed no change in mRNA expression (mean fold change 0.9 ± 0.34, *p* = 0.68) (Figure 1G) which indicated an intact IL-6/STAT3 signaling in HC and LNCaP cells but a disrupted signaling in HIES subjects and PC-3 cells.

### PCR Array Showed Differential Gene Expression Patterns in HCs, HIES Subjects, and Cell Lines

**Figure 2:**
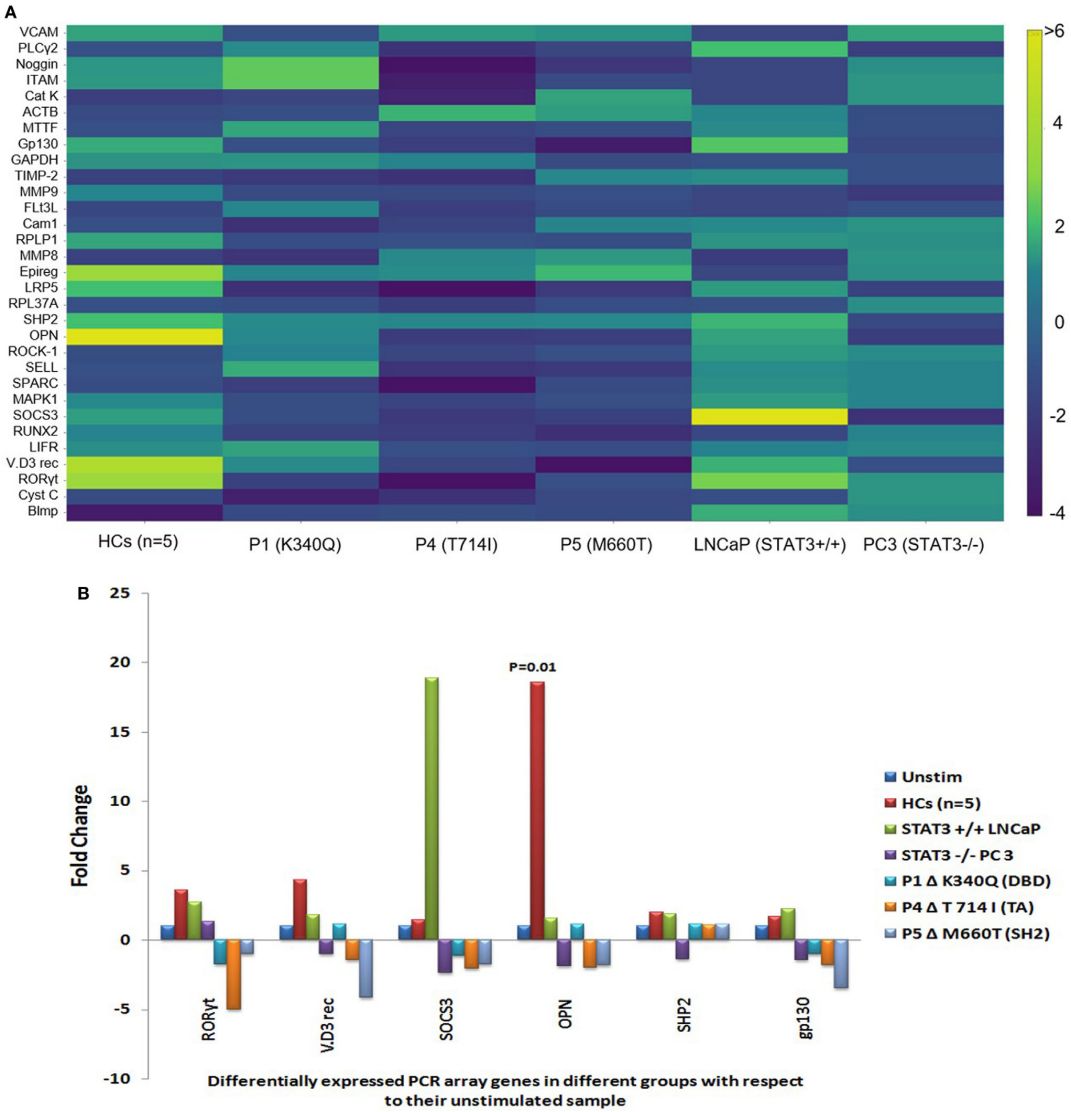
**(A)** Heat map of gene expression profile of HCs, hyper-IgE syndrome (HIES) subjects, LNCaP, and PC-3 cells generated using Plotly software (http/plot.ly). Scale bar −4 to +6 and >6. Fold change values were calculated using RT2 PCR array analysis software (Qiagen), an online tool. **(B)** Bar graphs showing differentially expressed genes in the PCR Array post IL-6 stimulation of HCs, LNCaP cells, PC3 cells, and HIES subjects with respect to their unstimulated samples. Data were normalized to mean *C*_t_ value of *β-actin, GAPDH*, and *RPL37A* genes. Statistical significance was determined by Student’s *t*-test (*p < 0.05, **p < 0.01, ***p < 0.001) using RT^2^ PCR Array Analysis software, Qiagen.

Of the 43 genes, PCR array in 12 genes showed *C*_t_ values beyond 35 and/or non-specific melting peaks and were hence excluded from analysis. Fold change difference in the remaining 31 gene sets in HCs (*n* = 5), HIES subjects, PC-3, and LNCaP is shown in the form of a heat map (Figure 2A). Differential fold change expression was observed in *OPN, RORyt, VitD3 receptor, SHP2, SOCS3*, and *gp130* genes between HCs and HIES subjects with respect to their respective unstimulated samples. *RORyt, VitD3 receptor, and SHP2* were found to be ≥2-fold upregulated in HCs, whereas *SOCS3* and *gp130* showed slight upregulation (mean fold change 1.5 and 1.7, respectively). However, PBMCs from HIES subjects showed either an unaltered expression or downregulation of these genes. *OPN* was found to be the most significantly upregulated gene in HCs (mean fold change 18.6, *p* = 0.01) and was not seen in HIES subjects: P1—unchanged, P4—2-fold downregulated, and P5—1.8-fold downregulated (Figure 2B). *OPN* was slightly upregulated in LNCaP cells (1.6-fold) and downregulated in PC-3 cells (−1.9-fold). *SOCS3* gene expression was significantly upregulated in LNCaP cells but not in PC-3 cells. Expression of *RORyt, VitD3 receptor, SHP2*, and *gp130* was found to be upregulated (≥2-fold) in LNCaP cells while it was either downregulated or unaltered in PC-3 cells (Figure 2B). Taken together, the PCR array results showed a set of 6 genes to be differentially upregulated of which OPN was the most prominent.

### *Stattic*-Induced Inhibition of STAT3 Suppresses OPN Gene Transcription

**Figure 3:**
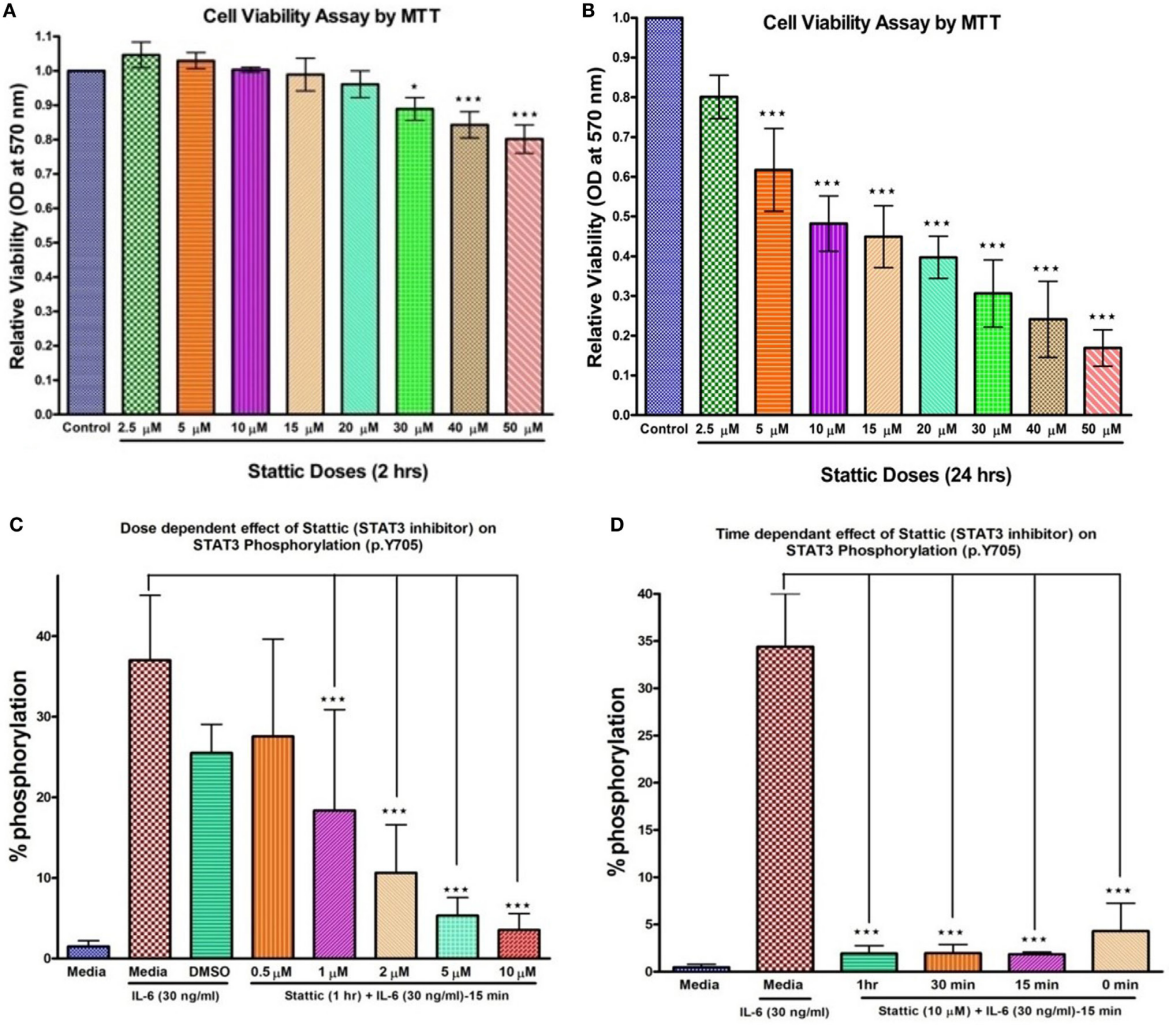
Bar graphs showing effect of *stattic* at different doses on cell viability and STAT3 phosphorylation on healthy controls peripheral blood mononuclear cells. Cell viability: **(A)** at 2 h, no effect on cell viability was observed except at high doses where growth inhibition was evident **(B)** at 24 h, growth was inhibited in a dose-dependent manner. *Stattic* abrogated the STAT3 activity in a dose **(C)** and time **(D)** dependent manner. Data are represented as mean ± SD for three independent experiments. *p < 0.05, **p < 0.01, ***p < 0.001, one way ANOVA post Tukey’s multiple comparison test.

To verify the PCR array findings, *OPN* gene expression was further validated by qRT-PCR in HCs, HIES subjects, LNCaP, and PC-3 stimulated with IL-6 for 2 h and the effect of *stattic* on *OPN* expression was studied in HC PBMCs. MTT assay was performed to assess toxicity of *stattic* on PBMC viability (Figures 3A,B). Dose and time optimization of *stattic* were done to assess its optimal effect on STAT3 phosphorylation by flow cytometry (Figures 3C,D). Maximum reduction in *STAT3* phosphorylation was observed at 10 µM concentration and hence 10 µM *stattic* for 1 h was chosen for the subsequent experiments.

**Figure 4:**
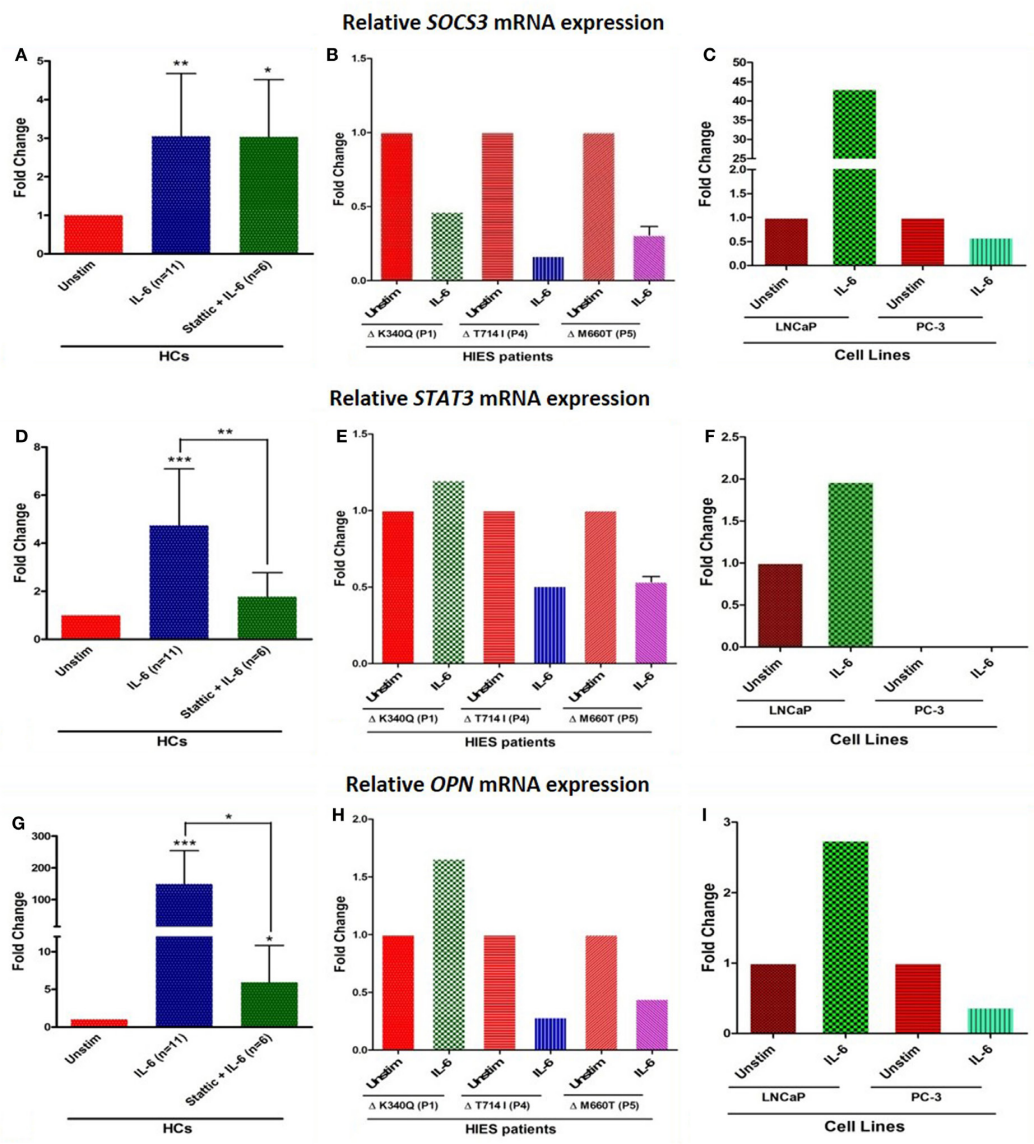
Bar graphs showing relative mRNA expression of *SOCS3, STAT3*, and *OPN* in HC peripheral blood mononuclear cells **(A,D,G)** treated with either IL-6 alone or with *stattic*; in hyper-IgE syndrome (HIES) subjects **(B,E,H)** treated with IL-6 alone and in LNCaP and PC-3 cells **(C,F,I)** treated with IL-6 alone. The primary transcript for each gene were assayed at least in duplicate and normalized to *β-actin* expression. Data represented as mean ± SD, *p < 0.05, **p < 0.01, ***p < 0.001, paired *t*-test and Wilcoxon test.

Relative mRNA expression of *SOCS3* and *STAT3* was found to be significantly upregulated in HCs after IL-6 treatment: mean fold change 3.0 ± 1.6 (*p* = 0.002) and 4.7 ± 2.3 (*p* = 0.0004), respectively, while HIES subjects showed either downregulated or unaltered mRNA expression. LNCaP cells showed 46- and 2-fold increase in *SOCS3* and *STAT3* expression while PC-3 cells showed 0.6-fold and no mRNA expression for these genes, respectively (Figures 4A–F). *Stattic*, however, failed to downregulate the *SOCS3* expression unlike *STAT3* and *OPN*. Further experiments to clarify this apparent discrepancy showed that this was because of a non-specific action of DMSO in which *stattic* was dissolved (Figure S1 in Supplementary Material). This effect was, however, not observed with *STAT3* or *OPN* mRNA expression. Careful scrutiny of studies looking at SOCS3 mRNA expression with DMSO as vehicle control, showed a significant upregulation of SOCS3 even with DMSO alone, and was more than empty vector/negative controls (29, 30). Diverse effects of DMSO on gene expression profiles have been documented in literature (31).

Osteopontin mRNA expression in HC showed up-regulation (mean fold change 149 ± 105, median 149, *p* = 0.001), whereas cells treated with *stattic* showed markedly diminished expression (mean fold change 6 ± 4.8, median 4, *p* = 0.02). HIES subjects showed 1.6-, 0.3-, and 0.4-fold change in *OPN* gene expression which was markedly reduced compared with HC. *OPN* expression in LNCaP and PC3 cells were 2.7- and 0.4-fold, respectively. LNCaP cells seemed to be somewhat deficient in OPN upregulation compared with HC PBMCs despite an intact STAT3 signaling (Figures 4G–I). Androgen-dependent LNCaP cells are known to express OPN to a much lesser extent compared with the androgen-independent counterparts like C4-2, and DU145 prostate cancer cell lines (32).

### Serum OPN Levels Do Not Correlate With STAT3 Induced OPN mRNA Expression

Mean serum *OPN* in HCs were 25 ± 9 ng/ml, whereas HIES subjects showed variable levels—3 (P1), 46 (P4), and 118 (P5) ng/ml. This observation suggests that serum *OPN in vivo* is regulated by pathways other than STAT3 and a defect in the IL-6/STAT3 pathway does not cause any apparent deficiency in serum *OPN* levels.

### *In Silico* Demonstration of STAT3 Response Element at Distal Enhancer Region of OPN Gene

**Figure 5:**
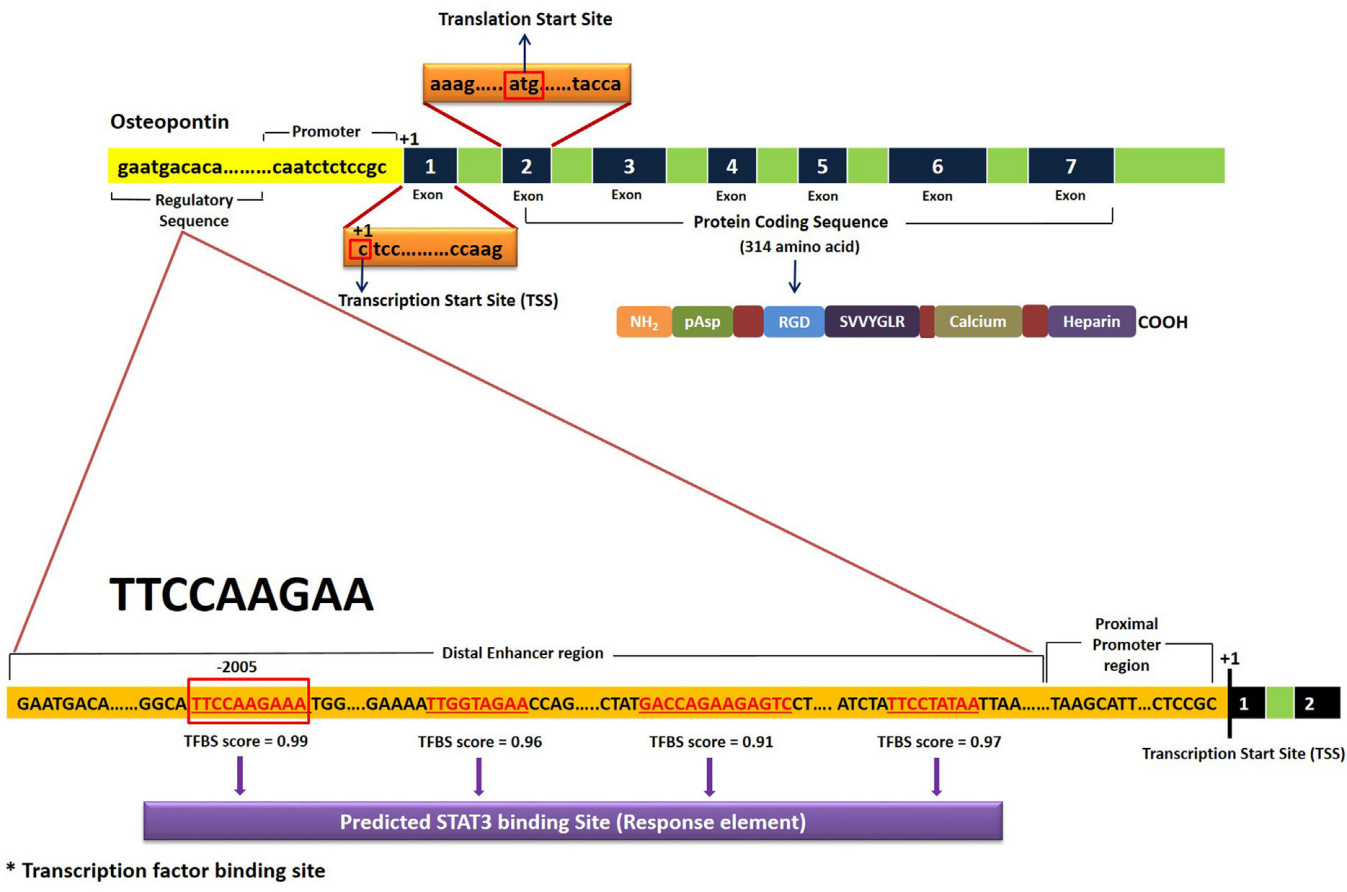
Schematic representation of STAT3 transcription factor binding site (TFBS) in the human OPN gene. Results were obtained using STAT Finder Bioinformatics Tool (33, 34).

Bioinformatic analysis to find a STAT3 transcription factor binding site (TFBS) in the OPN gene was done by YMO using the STAT finder Bioinformatic tool (33, 34), which predicted four response elements in the OPN gene with different binding scores (Figure 5). Consensus sequence TTCCAAGAA showed a maximum score of 0.99 and hence was considered to be the most likely TFBS for STAT3 in the human OPN gene. Similar response element with the consensus sequence TTCTGGGAA was observed in mice OPN gene with a score of 0.99.

## Discussion

Though both IL-6 and IL-11 signal through the same gp130 receptor β-subunit, and both activate STAT3, specificity is achieved through cell- and tissue-specific expression of the non-signaling α-receptor subunits. While IL6R is expressed in T-cells, monocytes, neutrophils, and pancreatic alpha cells, IL-11R is expressed in cardiac myocytes and endothelial and epithelial cells of the colon (35). In addition, IL-6/IL-6R is known to be the key signaling pathway in prostate cancer (36). Since we employed PBMCs and prostate cancer cells in the study, IL-6 was used as the stimulant. Our observations indicate that OPN gene expression in STAT3 LOF HIES in response to STAT3 activation is markedly diminished and the observation was supported by the PC3 and LNCap cell line data. Since cell lines are prone to genetic instability, they are not considered as good models for studying signaling pathways (37–39). Both LNCaP and PC3 cells used in the study were hence extensively characterized and validated with respect to STAT3 signaling before including them in the experiments. PCR array data pointed to a significant differential upregulation of *OPN* mRNA in HCs after IL-6 stimulation compared with HIES. Intriguingly enough, in the pre-STAT3 era, a marked under expression of *OPN* was observed in six of nine HIES subjects in the study by Chehimi et al. (40), 6 years before STAT3 was implicated in HIES. The finding, however, was ignored since the focus of the study was on traditional cytokine and chemokine dysregulation and TH1/TH2 imbalance in HIES. Sequencing of OPN gene from genomic DNA in that particular study did not reveal any mutations.

*Stattic* is known to potently and “selectively” inhibit STAT3 activation and nuclear translocation and selectively induce apoptosis of STAT3 dependent cancer cells (41–43). In our inhibition experiments using *stattic* in HC PBMCs, a downregulation of *OPN* gene expression was consistently observed indicating that *OPN* mRNA expression is induced upon IL-6 mediated STAT3 signaling. Though there is abundant literature on *OPN* regulation by different mechanisms (44–48), there was no emphasis in the literature on its regulation through STAT3 till a recent paper by Choi et al. which showed that OPN secreted by TM4SF4/GSK3β/β-catenin signaling activated the JAK2/STAT3 or FAK/STAT3 pathway which also upregulated OPN expression in an autocrine manner (49). Though *OPN* itself is known to induce STAT3 signaling (50, 51), its regulation through STAT3 pathway was a novel finding in our study, and is supported by Choi et al.’s study (49). This is further corroborated by a study that showed low mRNA expression of *OPN* in CD4+ve T cells conditionally knocked out for STAT3 gene (52). Our bioinformatic analysis predicted the presence of STAT3 response element at the distal enhancer region of *OPN* gene with consensus sequence TTCCAAGAA located at position-2005. This, however, needs to be further validated using reporter assay or chromatin immunoprecipitation assay.

*OPN* deficiency has been shown to suppress appearance of OdCs and resorption of tooth root induced by experimental force application (53). While wild-type mice showed appearance of OdCs around the mesial surface of the tooth root resulting in tooth root resorption, OPN knockout mice showed significantly suppressed force-induced increase in the number of OdCs and suppressed root resorption. Though this application of force also induced increase in the number of OCs in the alveolar bone on the pressure side, the number of OCs in such alveolar bone was similar between the *OPN*-deficient and wild-type mice which indicate that *OPN* deficiency specifically suppresses tooth root resorption by inhibiting the OdCs without affecting the adjacent bone OCs (53). The same study also showed reduced TNFα induced bone resorption using anti-OPN neutralizing antibody. Similar effects could be achieved using αvβ3 integrin receptor blockade using peptide inhibitor echistatin, an RGD containing peptide in a rat model (54, 55). The OdC numbers were, however, not affected implying that the blockade led to a functional defect in the OdCs. *OPN* is also known to be required for OC recruitment and RANKL expression during tooth drift-associated bone remodeling (56). Notably, OPN deficient mice show lower volume and length of OC ruffled borders, indicating lower resorptive capacity (57) despite normal numbers and also are hypomotile with reduced CD44 expression (58).

Though HIES subjects in our study showed low *OPN* mRNA expression, serum OPN levels were not reduced. This implied that the serum OPN was rescued in these patients apparently through other signaling pathways despite a defective STAT3-mediated OPN regulation. Although *OPN* does not seem to be required for skeletal development *per se*, and OPN knockout mice do not show any apparent skeletal malformation (59), its requirement in tooth resorption and cranial suture homeostasis might be a local site specific and time and age-dependent event which do not reflect on the general skeletal development. Moreover, not all patients of STAT3 LOF HIES show dental defects. The mediators of osteoclastogenesis (and osteoporosis) include many other factors like hormones (*viz*. estrogen, parathormone) and inflammatory cytokines (e.g., TNF-α, PGE2, VEGF, etc.). In contrast to alveolar bone of the skeleton which is rich in cells and blood vessels with abundance of these mediators, tooth cementum and dentin as well as cranial sutures are largely matrix dominant tissues and avascular. While the OPN deficiency in the trabecular bone of the skeleton can be easily overcome by the other mediators, the tooth cementum and cranial sutures may be largely dependent on the locally produced OPN by OdCs and OPN deficiency in this milieu may cause a state of deficiency. The osteoporosis observed in STAT3 LOF HIES on the other hand could be a result of the existence of a pro-inflammatory state rather than OPN deficiency.

## Conclusion

Together with *in vitro* and *in silico* experiments in the study, we concluded that STAT3 has a direct role in the transcriptional regulation of *OPN* gene and thus, STAT3 through *OPN*, might be involved in the development of dental/craniofacial manifestations observed in STAT3 LOF HIES. More detailed multi-centric collaborative studies need to be performed in larger STAT3 LOF HIES cohorts with and without dental/facial manifestations using other appropriate biological systems like primary OCs/OdCs and macrophages to completely understand its exact role.

## Ethics Statement

Institute Ethics Committee (IEC No. 8827-PG 10-1TRG/8450). All subjects were recruited after obtaining an informed consent prior to enrolment as per IEC guidelines.

## Author Contributions

SG participated in performance of research, data analysis, and writing of paper; SS and DS treated the patients; SmS did the flow cytometry experiments; RM, ShS, and AR participated in writing of the manuscript; YO did the bioinformatics analysis; BS participated in research design, result interpretation, and writing of manuscript.

## Conflict of Interest Statement

The authors declare that the research was conducted in the absence of any commercial or financial relationships that could be construed as a potential conflict of interest.
