# Supplementary material for: STAT3-Mediated Transcriptional Regulation of Osteopontin in STAT3 Loss-of-Function Related Hyper IgE Syndrome
Source: Front Immunol. 2018 May 17;9:1080. doi: 10.3389/fimmu.2018.01080 (PMC5966547; doi:10.3389/fimmu.2018.01080)
Supplement: Supplementary file 1 [file Image_1.PDF]

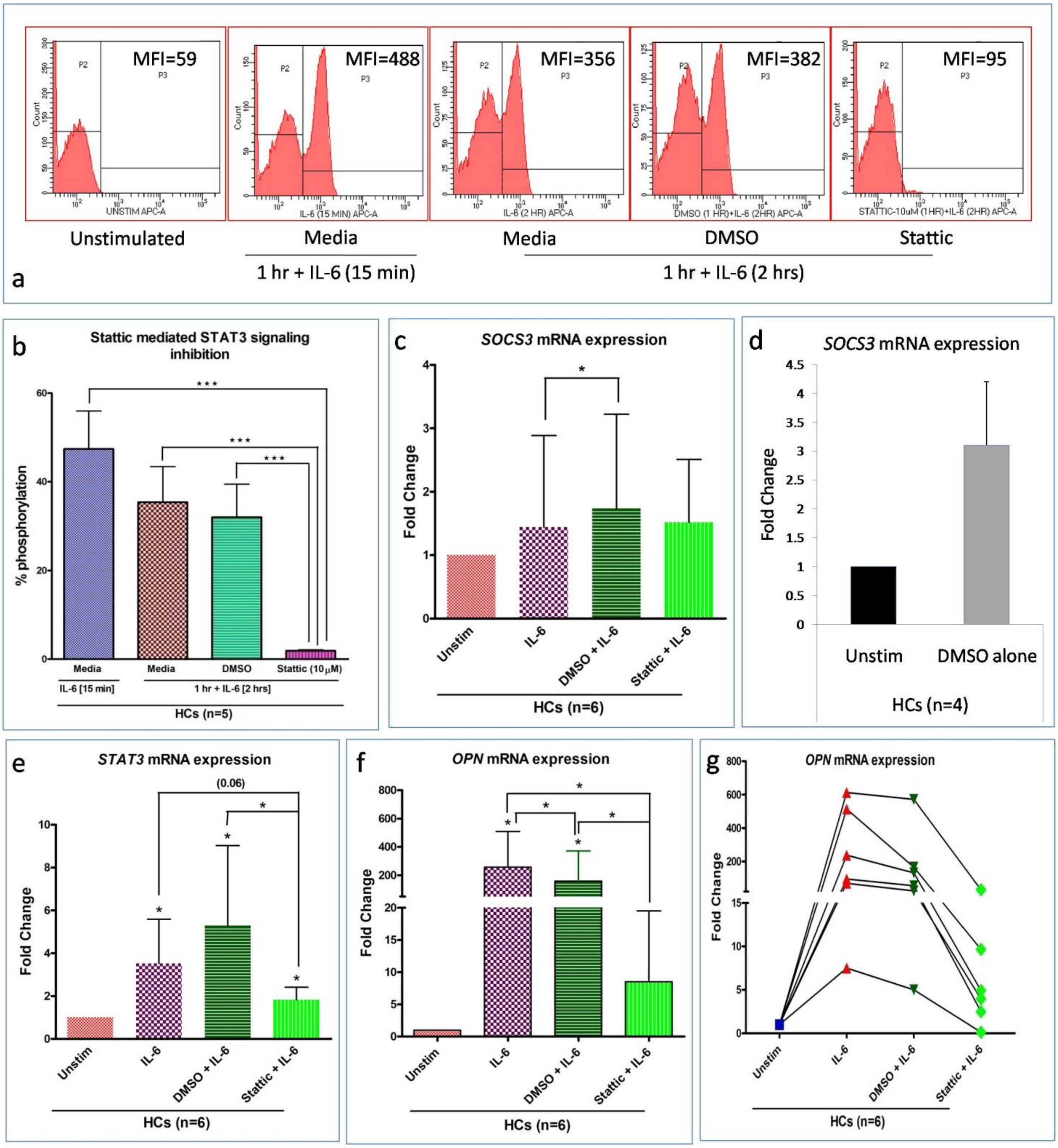

Supplementary Figure 1. Since cells from HCs treated with *stattic* failed to show downregulation of *SOCS3* mRNA (mean fold change 3.0 ± 1.5, p=0.01), as shown in **figure 4a** and was not expected, we sought to rule out the effect of residual IL-6 activity and/or nonspecific action of DMSO (in which *stattic* was reconstituted) in upregulation of *SOCS3*. Assays were done to confirm whether the duration of IL-6 treatment (15 minutes versus 2 hours) and/or presence of DMSO altered the STAT3 phosphorylation i.e. STAT3 activity. **a.**

Representative histograms and **b**. bar graphs showing pSTAT3 activity of cells treated with media, DMSO and *stattic* in the presence of IL-6. No difference was observed in pSTAT3 with either media or DMSO and there was effective inhibition of STAT3 activity by *stattic*, ruling out the effect of DMSO or residual IL-6 activity on pSTAT3. **c**. Cells treated with IL-6 + DMSO showed more upregulation of *SOCS3* mRNA expression compared to IL-6 alone (p=0.03) indicating an additional effect of DMSO. Results also indicated that the failure of *stattic* to downregulate *SOCS3* was restricted only to *SOCS3* and was not seen with STAT3. **d.** This upregulation of *SOCS3* was more importantly also observed with DMSO alone (without IL-6) indicating a non-specific effect of DMSO on upregulation of *SOCS3*. **e**. This effect of DMSO was also seen with *STAT3* mRNA expression where IL-6 + DMSO showed a greater upregulation of *STAT3* mRNA expression compared to IL-6 alone, but this was effectively downregulated by *stattic*. **f**. mRNA expression of OPN was significantly increased on stimulation with IL-6 both with and without DMSO and this was significantly reduced with *stattic*, though not up to the extent of basal unstimulated cells. The non-specific effect of DMSO+IL-6 pver IL-6 alone was not observed in OPN expression unlike SOCS3 and STAT3. **g**. *OPN* fold change values, when represented in the form of individual line graphs clearly showed a trend that each individual had upregulation in *OPN* mRNA expression after treatment with IL-6 alone with or without DMSO and expression was decreased on treatment with *stattic*.
