# Supplementary material for: STAT3-Mediated Transcriptional Regulation of Osteopontin in STAT3 Loss-of-Function Related Hyper IgE Syndrome
Source: Front Immunol. 2018 May 17;9:1080. doi: 10.3389/fimmu.2018.01080 (PMC5966547; doi:10.3389/fimmu.2018.01080)
Supplement: Supplementary file 2 [file Table_1.PDF]

Supplementary table 1: List of genes studied in the PCR array with their alternate names and function.

Gene Symbol	Gene Name	Alternate Name	Gene Function
**Blmp1**	B Lymphocyte-induced Maturation Protein-1		A transcriptional repressor important for the differentiation and function of several types of immune cells. Blimp-1 negatively regulates several cytokine/chemokine genes.
**Cyst C**	Cystatin C	ARMD11	Active cysteine protease inhibitors. A mutation in this gene has been associated with amyloid angiopathy and age-related macular degeneration.
**LIF**	Leukemia inhibitory factor		LIF, is an interleukin 6 class cytokine that affects cell growth by inhibiting differentiation.
**Onco M**	Oncostatin M		Belongs to the interleukin 6 group of cytokines. It is also associated with bone formation and destruction.
**RORyt**	RAR-related orphan receptor gamma/C	*RORC*	DNA-binding transcription factor and involved in the regulation of circadian rhythms. It also plays a role in inhibiting apoptosis of undifferentiated T cells and promoting their differentiation into Th17 cells.
**V.D3 rec**	Vitamin D (1,25-dihydroxyvitaminD3) receptor	NR1I1 or calcitriol receptor	Downstream targets of this nuclear hormone receptor are principally involved in mineral metabolism though the receptor regulates a variety of other metabolic pathways, such as those involved in the immune response and cancer. Mutations in this gene are associated with type II vitamin D-resistant rickets.
**BSP**	Bone sialoprotein	IBSP, integrin-binding sialoprotein	Major structural protein of the bone matrix. This protein binds to calcium and hydroxyapatite and mediates cell attachment. Affect bone mineral density and fracture risk. Additional roles of BSP are MMP-2 activation, angiogenesis, and protection from complement-mediated cell lysis.
**DKK1**	Dickkopf WNT signaling pathway inhibitor 1	Dickkopf-related protein 1	It is a secreted protein with two cysteine rich regions and is involved in embryonic development through its inhibition of the WNT signaling pathway. Elevated levels of DKK1 in bone marrow plasma and peripheral blood is associated with the presence of osteolytic bone lesions in patients with multiple myeloma.
**LIFR**	Leukemia inhibitory factor receptor alpha	CD118 (Cluster of Differentiation 118)	Belongs to the type I cytokine receptor family.apolyfunctional cytokine that is involved in cellular differentiation, proliferation and survival in the adult and the embryo. Mutations in this gene cause Schwartz-Jampel syndrome type 2, a disease belonging to the group of the bent-bone dysplasias.
**BGP**	Bone gamma-carboxyglutamic acid-containing protein	Osteocalcin, BGLAP	A noncollagenous protein found in bone and dentin. Implicated in bone mineralization and calcium ion homeostasis.
**RUNX2**	Runt -related transcription factor 2	Core binding factor subunit alpha-1	Essential for osteoblastic differentiation and skeletal morphogenesis. Mutation cause Cleidocranialdysostosis where there is delayed ossification of midline structure
**SOCS3**	Suppressor of cytokine signaling 3		STAT-induced STAT inhibitor (SSI).negative regulators of cytokine signaling. bind to JAK2 kinase, and inhibit the activity of JAK2 kinase.
**b3 int**	Integrin beta-3 or platelet glycoprotein IIIa	CD61, ITGB3	Integrin beta 3 is found along with the alpha IIb chain in platelets. Integrins are known to participate in cell adhesion as well as cell-surface mediated signalling.

**DKK2**	Dickkopf WNT signaling pathway inhibitor 2		It can act as either an agonist or antagonist of Wnt/beta-catenin signaling, depending on the cellular context and the presence of the co-factor kremen 2.
**MAPK1**	Mitogen-activated protein kinase 1	ERK (extracellular signal-regulated kinases)	Involved in a wide variety of cellular processes such as proliferation, differentiation, transcription regulation and development
**SPARC**	Secreted protein acidic and rich in cysteine (Osteonectin)	Basement-membrane protein 40 (BM-40)	The encoded protein is required for the collagen in bone to become calcified but is also involved in extracellular matrix synthesis and promotion of changes to cell shape. Osteonectin also increases the production and activity of matrix metalloproteinases
**SELL**	Selectin L		Belongs to a family of adhesion/homing receptors. Required for binding and subsequent rolling of leucocytes on endothelial cells, facilitating their migration into secondary lymphoid organs and inflammation sites.
**ROCK-1**	Rho-associated, coiled-coil containing protein kinase 1		Serine/threonine kinase that is activated when bound to the GTP-bound form of Rho. The small GTPase Rho regulates formation of focal adhesions and stress fibers of fibroblasts, as well as adhesion and aggregation of platelets and lymphocytes
**Calcen**	Calcineurin	Protein phosphatase 3 PPP3CA	Calcium-dependent serine-threonine phosphatase. Calcineurin activates nuclear factor of activated T cell, cytoplasmic (NFATc), a transcription factor, by dephosphorylating it. The activated NFATc is then translocated into the nucleus, where it upregulates the expression of interleukin 2 (IL-2), which, in turn, stimulates the growth and differentiation of T cells.
**Epigen**	Epithelial mitogen	EPGN	Members of this family are ligands for the epidermal growth factor receptor and play a role in cell survival, proliferation and migration. This protein has been reported to have high mitogenic activity but low affinity for its receptor.
**MMP2**	Matrix metallopeptidase 2	72 kDa type IV collagenase, gelatinase A	Involved in the breakdown of extracellular matrix in normal physiological processes, such as embryonic development, reproduction, and tissue remodeling, Mutations in this gene have been associated with Winchester syndrome and Nodulosis-Arthropathy-Osteolysis (NAO) syndrome.
**OPN**	Osteopontin	SPP-1 (Secreted phosphoprotein1)	Involved in the attachment of osteoclasts to the mineralized bone matrix. Upregulates expression of interferon-gamma and interleukin-12
**SHP2**	Protein tyrosine phosphatase, non-receptor type 11	PTPN11	Regulate a variety of cellular processes including cell growth, differentiation, mitotic cycle, and oncogenic transformation. Mutations in this gene are a cause of Noonan syndrome as well as acute myeloid leukemia.and LEOPARD syndrome 1 and have also been associated with Metachondromatosis.
**LRP5**	Low density lipoprotein receptor-related protein 5		Binds and internalizes ligands in the process of receptor-mediated endocytosis. Also acts as a co-receptor with Frizzled protein family members for transducing signals by Wnt proteins. Plays a key role in skeletal homeostasis and many bone density related diseases. Mutation cause familial exudative vitreoretinopathy
**Epireg**	Epiregulin	EREG	Member of the epidermal growth factor family. Epiregulin can function as a ligand of EGFR (epidermal growth factor receptor), as well as a ligand of most members of the ERBB (v-erb-b2 oncogene homolog) family of tyrosine-kinase receptors.

**MMP8**	Matrix metallopeptidase 8 (neutrophil collagenase)		Involved in the breakdown of extracellular matrix in normal physiological processes, such as embryonic development, reproduction, and tissue remodeling, as well as in disease processes, such as arthritis and metastasis.
**Ostprot**	Osteoprotegerin (OPG) or osteoclastogenesis inhibitory factor (OCIF),	Tumor necrosis factor receptor superfamily member 11B (TNFRSF 11B)	An osteoblast-secreted decoy receptor that functions as a negative regulator of bone resorption. Mutation is associated with Hyperphosphatasemia with bone disease (Juvenile Paget disease).
**SIRT-1**	Sirtuin 1	NAD-dependent deacetylase sirtuin-1	The functions of human sirtuins have not yet been determined; however, yeast sirtuin proteins are known to regulate epigenetic gene silencing and suppress recombination of rDNA. Human sirtuins may function as intracellular regulatory proteins with mono-ADP-ribosyltransferase activity.
**Cam1**	Calmodulin (CALcium-MODULatedproteIN)		CaM mediates many crucial processes such as inflammation, metabolism, apoptosis, smooth muscle contraction, intracellular movement, short-term and long-term memory, and the immune response. CaM is expressed in many cell types
**FLt3L**	fms-related tyrosine kinase 3 ligand		FLT3LG controls the development of DCs and is particularly important for plasmacytoid DCs and CD8
**MMP9**	Matrix metallopeptidase 9 (gelatinase B)		Involved in the breakdown of extracellular matrix in normal physiological processes. The enzyme encoded by this gene degrades type IV and V collagens. Mutation cause Metaphysealanadysplasia 2
**Osterix**	Sp7 transcription factor	SP7, Osterix	This protein is a bone specific transcription factor and is required for osteoblast differentiation and bone formation. Mutation Cause Osteogenesisimperfecta type 11.
**TIMP-2**	TIMP metallopeptidase inhibitor 2		Natural inhibitors of the matrix metalloproteinases, a group of peptidases involved in degradation of the extracellular matrix. Associated with Wntsignaling and Dupuytren's disease
**Cat E**	Cathepsin E		An Intracellular aspartic proteinase expressed predominantly in immune cells and skin. Cathepsin E deficient mice have been observed to spontaneously develop features of Atopic Dermatitis very similar to human AD with elevated IgE and eosinophilia
**gp130**	Glycoprotein 130	IL6ST (interleukin 6 signal transduce), IL6-beta or CD130	Signal transducer shared by many cytokines, including interleukin 6 (IL6), ciliaryneurotrophic factor (CNTF), leukemia inhibitory factor (LIF), and oncostatin M (OSM). Knockout studies in mice suggest that this gene plays a critical role in regulating myocyte apoptosis.
**MTTF**	Mitochondrially encoded tRNAphenylalanine	TRNF (it is RNA not a protein)	Mutation cause Juvenile myopathy, encephalopathy, lactic acidosis AND stroke.
**PIAS3**	Protein inhibitor of activated STAT3	E3 SUMO-protein ligase PIAS	The protein functions as a SUMO (small ubiquitin-like modifier)-E3 ligase which catalyzes the covalent attachment of a SUMO protein to specific target substrates. It directly binds to several transcription factors and either blocks or enhances their activity.
**TWIST1**	Twist family bHLH transcription factor 1		Basic helix-loop-helix (bHLH) transcription factors have been implicated in cell lineage determination and differentiation. Mutations in this gene have been found in patients with Saethre-Chotzen syndrome. Craniosynostosis 1 and RobinowSorauf syndrome
**Cat K**	Cathepsin K		Lysosomal cysteine proteinase involved in bone remodeling

			and resorption. Mutations in this gene are the cause of pycnodysostosis, an autosomal recessive disease characterized by osteosclerosis, dental anomalies and short stature.
**T3 zeta chain**	**T-cell surface glycoprotein CD3 zeta chain**	**CD247**	T-cell receptor zeta, together with T-cell receptor alpha/beta and gamma/delta heterodimers and CD3-gamma, -delta, and -epsilon, forms the T-cell receptor-CD3 complex. The zeta chain plays an important role in coupling antigen recognition to several intracellular signal-transduction pathways. Low expression of the antigen results in impaired immune response. Defect in CD3-zeta cause eosinophilia, Immunodeficiency, Protracted diarrhea, Susceptibility to herpesvirus and T lymphocytopenia
**Noggin**	Noggin	NOG	Secreted polypeptide binds and inactivates members of the transforming growth factor-beta (TGF-beta) superfamily signaling proteins, such as bone morphogenetic protein-4 (BMP4). Several dominant human NOG mutations in unrelated families with proximal symphalangism (SYM1) and multiple synostoses syndrome (SYNS1) were identified; both SYM1 and SYNS1 have multiple joint fusions as their principal feature.
**PLCy2**	Phospholipase C, gamma 2 (phosphatidylino-sitol-specific)	CD106	Catalyzes the conversion of 1-phosphatidyl-1D-myo-inositol 4,5-bisphosphate to 1D-myo-inositol 1,4,5-trisphosphate (IP3) and diacylglycerol (DAG) using calcium as a cofactor. Mutations in this gene have been found in autoinflammation, antibody deficiency, and immune dysregulation syndrome and familial cold autoinflammatory syndrome 3.
**VCAM**	Vascular cell adhesion molecule 1		Sialoglycoprotein expressed by cytokine-activated endothelium. This type I membrane protein mediates leukocyte-endothelial cell adhesion and signal transduction, and may play a role in the development of artherosclerosis and rheumatoid arthritis.
**RPL37A**	Ribosomal protein L37a (house keeping gene)	L37A	Ribosomal protein that is a component of the 60S subunit.contains a C4-type zinc finger-like domain. (House keeping gene)
**RPLP1**	60S acidic ribosomal protein P1 (housekeeping gene)	Ribosomal protein, large, P1	This gene encodes a ribosomal phosphoprotein that is a component of the 60S subunit. (House keeping gene)
**GAPDH**	Glyceraldehyde-3-phosphate dehydrogenase (housekeeping gene)		Involved in oxidative phosphorylation of glyceraldehyde-3-phosphate in the presence of inorganic phosphate and nicotinamide adenine dinucleotide (NAD). (House keeping gene)
**ACTB**	Beta actin (house keeping gene)		This is one of the two nonmuscle cytoskeletal actins. Actins are highly conserved proteins that are involved in cell motility, structure and integrity. (House keeping gene)
